# Novel composite clinical endpoints and risk scores used in clinical trials in pulmonary arterial hypertension

**DOI:** 10.1177/2045894020962960

**Published:** 2020-11-18

**Authors:** Olivier Sitbon, Sylvia Nikkho, Raymond Benza, Chunqin (CQ) Deng, Harrison W. Farber, Mardi Gomberg-Maitland, Paul Hassoun, Christian Meier, Joanna Pepke-Zaba, Krishna Prasad, Werner Seeger, Paul A Corris

**Affiliations:** 1 Assistance Publique–Hôpitaux de Paris, Service de Pneumologie, Hôpital Bicêtre, Université Paris-Sud, Laboratoire d’Excellence en Recherche sur le Médicament et Innovation Thérapeutique, Le Kremlin-Bicêtre, France; 2 1569 Bayer AG, Clinical Development, Berlin, Germany; 3 The Ohio State University, Columbus, OH, USA; 4 17909 United Therapeutics Corporation, Raleigh-Durham, NC, USA; 5 Tufts Medical Center, Boston, MA, USA; 6 8367 George Washington University of Medicine and Health Sciences, Washington, DC, USA; 7 1500 Johns Hopkins University School of Medicine, Baltimore, MD, USA; 8 Royal 2144 Papworth Hospital NHS Foundation Trust, Cambridge, UK; 9 Medicines and Healthcare Products Regulatory Agency (MHRA), London, UK; 1 ^0^University of Giessen and Marburg Lung Center (UGMLC), Giessen, Germany; 1 ^1^Clinical and Translational Research Institute Newcastle University, Newcastle upon Tyne, UK

**Keywords:** drug development, pulmonary arterial hypertension, risk factors, risk stratification and biomarkers

## Abstract

This manuscript on endpoints incorporates the broad experience of members of Pulmonary Vascular Research Institute’s Innovative Drug Development Initiative as an open debate platform for academia, the pharmaceutical industry and regulatory experts surrounding the future design of clinical trials in pulmonary hypertension. It reviews our current understanding of endpoints used in phase 2 and 3 trials for pulmonary hypertension and discusses in detail the value of newer approaches. These include the roles of composite endpoints and how these can be developed and validated. The newer concept of risk analysis is also discussed, including how such risk scores might be utilised as endpoints in clinical trials.

## Take home messages

Future clinical trials of novel therapeutic approaches in pulmonary hypertension (PH) will require consideration of new endpoints that are commensurate with a robust meaningful result in the face of shorter duration trials and the trial population already receiving multiple background treatments.

Validated composite endpoints which reflect long-term morbidity and mortality are essential and should ideally include measures that patients have indicated as important. There is a clear need to base endpoints on clinical improvement rather than a delay in clinical worsening (CW).

A change in risk scores may well be utilised in the future, and important to note that these indices should be well balanced in both arms of the trial population at randomisation given the impact of starting risk on outcome.

## Introduction

Pulmonary arterial hypertension (PAH) is a serious chronic disease resulting in reduced life expectancy despite available medications.^1–4^ There is an urgent need to develop new drug therapies into routine clinical practice, hence requiring the development of novel study designs, including new endpoints. These endpoints will need to be meaningful to patients, physicians and regulators, as well as practical entities, in order to enable such trials to become feasible.

PAH patients suffer from dyspnoea, hypoxemia, heart failure and muscle weakness, which lead to limited physical activity. It has been reported that the decline in physical activity directly impacts patients’ daily performance and health-related quality of life (HRQoL). Limitations in physical activity impact emotional, social and daily life aspects, such as struggling to perform basic household tasks or caring for family and children. It is important to improve this level of physical activity in patients suffering from PH, as it has a direct impact on overall health, well-being and quality of life.^5^

The focus of this manuscript on novel endpoints is based on the considerable experience gained in PAH. The in-depth risk score assessment undertaken may model approaches for other types of PH in the future. There is a need to identify novel trial design and endpoints if we are going to meet patients’ expectations for meaningful outcomes and feasible study design. This is also important in patients with PH associated with pre-existing cardiovascular or chronic respiratory disease, as the long-term outcomes remain similarly poor as those for PAH.^6^

Therefore, based on a review of established endpoints used in clinical trials and risk scores used in registries, proposals of potential approaches including discussion of limitations and next steps are presented.

## Single improvement endpoints

Traditional single improvement endpoints have been used in PAH trials covering haemodynamics, e.g. pulmonary vascular resistance (PVR), for phase 2 studies, or exercise capacity, e.g. six-minute walking distance (6MWD), for phase 3 studies. However, endpoints should be tailored to the disease biology and anticipated mechanistic effects in a comprehensive way.^7,8^

Phase 2 studies in PH have frequently used invasive haemodynamics, particularly PVR as a primary endpoint and this as a measure of a new drug’s efficacy is likely to remain a valued measure especially until a newer biomarker of damage to the pulmonary vasculature may become validated.^7,9^

Single primary endpoints, measuring improvement in exercise capacity, such as a short-term change in the 6MWD, were introduced for phase 3 studies in the 1990s.^9^ However, it is widely recognised that these have limitations for use in clinical trials in PAH,^10^ mainly because many such variables do not clearly associate with patient-relevant outcomes like mortality and morbidity.^11^ Moreover, while increase in 6MWD was sensitive to change in the single agent placebo-controlled trials of the past, there is much less confidence in using this single primary endpoint in future trials where combined therapy is often the norm.

One relatively new endpoint reflecting clinical improvement or deterioration of direct relevance to PAH patients involves the use of continuous activity monitoring devices. Data from physical activity monitoring, such as actigraphy, have been collected and used in a variety of patient populations for decades. Specifically, in cardiac and pulmonary diseases, these studies have helped establish the value of directly measuring physical activity based on correlations with other measures of clinical improvement, such as survival, disease severity and quality of life. Generally, a change in moderate activity of 10–20% has been clinically relevant in cardiopulmonary diseases.^12,13^ Small-scale studies in PAH/chronic thromboembolic pulmonary hypertension (CTEPH) patients indicated that a long nocturnal rest and reduced daytime activity recorded by actigraphy were associated with severe haemodynamic impairment and reduced survival in patients with PH.^14^ Using the Actigraph device, treatment with inhaled nitric oxide in PH due to lung fibrosis demonstrated, in the first cohort of a phase 2/3 study, improvement in moderate/vigorous physical activity, as well as in overall activity.^15^

However, further validation and standardisation of different methods used are needed for the PAH population; these may be forthcoming in larger scale randomised controlled trials (RCTs), such as the phase 4 TRACE study evaluating the effect of selexipag on daily life physical activity and on patient-reported disease symptoms in PAH.^16^ A clinically meaningful difference should be determined and predictivity for long-term outcome demonstrated for future studies in the respective target population.^9^

## Composite clinical endpoints

### Time to clinical worsening

A composite endpoint looking at prevention of disease progression, time to clinical worsening (TTCW), has increasingly been used as a primary endpoint to evaluate treatment differences in disease progression. However, despite a better correlation between outcomes, the use of these types of endpoints has led to longer and larger studies with incurrent increases in study cost. Based on analogous endpoint in heart failure trials, worsening of heart failure or hospitalisation for heart failure, both the FDA and the European Medicines Agency continue to encourage the use of TTCW as a composite primary endpoint in PAH clinical trials.^9^

TTCW has been used in five pivotal clinical trials in PAH,^17–21^ and is often referred to as a ‘morbidity/mortality’ endpoint, although mortality has never accounted significantly for the difference between treatment arms in those trials. McLaughlin et al.^22^ noted, in the 2009 Dana Point recommendations, that the components of TTCW were variably applied in clinical trials. Indeed, although this endpoint was designed to comprehensively analyse clinical deterioration, inconsistencies between the specific components make trial comparisons difficult. In addition, event-driven trials with TTCW as a primary endpoint require relatively long study durations and large patient numbers.^8^ Demonstrating a survival benefit appears unfeasible in clinical trials with a small number of mortality events in a rare disease compared with large heart failure trials.

The introduction of potential new effective PAH therapies will require trials using patients receiving background PAH therapies, thus, demonstrating efficacy in terms of survival in an add-on study design will prove even more challenging.

Recent experience from the AMBITION-like study design, with TTCW endpoint applied, indicated that the event rate becomes lower, and thereby required larger patient cohorts followed over a long period of time to show difference. This may be caused by a large proportion of the inclusion of low-risk patients on combination therapy. Enrichment strategies to include intermediate- to high-risk patients may overcome unfeasible trial sizes.

There is a measurable burden to PAH patients taking part in a lengthy trial, which may or may not be positive. In general, however, patients who enter clinical trials have better outcomes in real world conditions than those treated outside of a clinical trial. Clearly, there is an urgent need to identify and validate more sensitive, reliable, and practical study endpoints and their responses to therapy.^8,23^ The use of enrichment strategies at study entry, as discussed above, with relevant prognostic risk scores would seem important to consider for future trials.

### Combined clinical improvement endpoints used in clinical trials

The components of any proposed combined endpoint with the highest discrimination potential at follow-up must be utilised to allow for confidence in the simulated or predicted clinical outcome.^7^ Thus, there is a movement to develop novel combined endpoints utilising collections of known risk variables that are more sensitive and more accurate in predicting eventual mortality and morbidity events. The advantage of combining different variables in such an endpoint is that the overall event rate increases and thereby the number of patients needed may be reduced for a trial. These combinations of variables are also incorporated in contemporary risk scores and risk calculators.

Incorporating clinical paradigms in PAH that reflect improvement in a patient’s physical capacity and/or risk status, that could be utilised in future clinical studies to ensure reasonable sample size and duration, stimulated the discussion on novel primary endpoints of clinical improvement.^23^ A good clinical endpoint should measure a relevant improvement or deterioration of the clinical condition over the shortest period. The composite endpoint of clinical improvement and/or an index of risk score could be applied in clinical trials as measures to demonstrate the clinical benefit in improvement and in lowering the risk score. Also, from the patient perspective, it may be more relevant to have an endpoint to measure the rapidity of improvement in physical capacity and well-being than waiting for progression of this life-threatening disease. A challenge to the use of composite endpoints in future studies will be that many patients are receiving double and triple combination therapy. Therefore, it needs to be proven whether composite endpoints of clinical improvement open new perspectives in contemporary studies investigating efficacy or treatment standards in double and even triple combination therapy.

While, in the 1990s, the majority of PAH studies starting with prostacyclin used the 6MWD as a primary endpoint,^24–28^ a composite endpoint of clinical improvement was selected, as the primary endpoint, for the pivotal phase 3 study with inhaled iloprost over a 12-week period (AIR study). This was the first combination of clinically relevant and predictive components, such as the improvement of functional class (FC) and 6MWD in the absence of deterioration which allowed for a more rigorous assessment of efficacy in each patient and led to regulatory approval of inhaled iloprost in 2003.^29^

Studies that have included composite endpoints of clinical improvement are as follows:

#### AIR study – a phase 3 double-blinded placebo-controlled randomised two-arm study

This 12-week study of inhaled iloprost enrolled a total of 203 New York Heart Association Functional Class (NYHA FC) III and IV patients, with primary pulmonary hypertension and non-primary forms of PH. The forms of non-primary PH included appetite suppressant-related and scleroderma-associated PAH, as well as inoperable CTEPH. The primary endpoint of the study consisted of an increase of at least 10% in the 6MWD and an improvement in the NYHA FC in the absence of clinical deterioration or death during the 12 weeks of the study.^29^ A significant treatment effect in favour of iloprost versus placebo (*P* = 0.007) with an estimated odds ratio of 3.97 (95% CI: 1.47–10.75) was found at 12 weeks. Nearly 40% of patients showed increased 6MWD by at least 10%. Approximately 20% of patients showed improvement in FC. However, not all patients with improved FC had a 10% increase in 6MWD. Therefore, the responder rate appeared rather small with 16.8% in the iloprost and 4.9% in the placebo group.^29^

#### AMBITION study – an event-driven, double-blinded randomised phase IV study

The study included 500 treatment-naïve PAH patients with the World Health Organization (WHO) FC II or III randomised to receive upfront once daily combination therapy with 10 mg of ambrisentan plus 40 mg of tadalafil (combination therapy group), 10 mg of ambrisentan plus placebo (ambrisentan monotherapy group) or 40 mg of tadalafil plus placebo (tadalafil monotherapy group). The primary endpoint in a time-to-event analysis was the first predefined event of clinical failure (defined as the first occurrence of a composite of death, hospitalisation for worsening PAH, disease progression or unsatisfactory long-term clinical response). A key secondary endpoint, according to the pre-specified hierarchical testing procedure, was the percentage of participants with a satisfactory clinical response. This was defined as an increase of 10% from baseline in the 6MWD, with a reduction to, or maintenance of, the WHO FC class I or II and no events of worsening clinical condition before or at the week 24 visit.^19^ In contrast to the AIR study, in the AMBITION study, the criteria were adjusted to assess patients already in FC II at baseline. Here, the requirement was to improve to FC I or II, or to maintain in FC II. This may have resulted in a higher rate of patients with satisfactory clinical response as the upfront combination therapy group was 39% (pooled monotherapy group 29%) at 24 weeks.^19,30^

#### RESPITE study – a 24-week, prospective, exploratory, open-label, multicentre, uncontrolled, single-arm study

In this study, 61 patients with symptomatic PAH in WHO FC III, who were not clinically improved with phosphodiesterase-5 inhibitor (PDE-5i) mostly in combination with ERAs, had the PDE-5i switched to riociguat. As an exploratory endpoint, the study applied a composite responder endpoint of freedom from CW, improvement of 6MWD ≥ 30 m and improvement to the WHO FC I/II. In the WHO FC III PAH patients on stable PDE-5i treatment at baseline, 16/51 (31%) reached the responder endpoint at 24 weeks after switching to riociguat.^31^

#### REPLACE study – a prospective, randomised-controlled, multicentre, 24-week open-label two-arm phase IV study

This study included 221 patients and compared replacement of PDE-5i treatment by riociguat against maintenance of PDE-5i maintenance in stable PAH patients on PDE-5i with or without ERA but not at treatment goal (WHO FC III). The primary endpoint of SCR is improvement of 6MWD by ≥10% or ≥30 m, improvement to FC I–II and decrease in N-terminal prohormone of brain natriuretic peptide (NT-proBNP) by at least 30% (two of three of these satisfactory clinical responses must be fulfilled) in the absence of CW.^32^ In order to investigate the discriminating power and relevance for long-term clinical outcome, the endpoint was applied post-hoc to the pivotal riociguat study database (PATENT-1/2).^33^ The results of the REPLACE study are to be presented by the end of 2020.

### Risk scores used in registries

Previous large-scale registry analyses of risk scores have identified improvement of invasive (cardiac index, right atrial pressure, SvO_2_) and/or non-invasive (FC, 6MWD and brain natriuretic peptide (BNP)/NT-proBNP, vital signs, renal function) variables to be related to long-term outcomes of patients with PAH.^34–37^ In addition, risk scores were used in exploratory analyses of randomised, controlled clinical trials; from these trials, it appeared that they may be sufficiently sensitive to differentiate the treatment effects of an experimental therapy.^21,38–40^

A meeting of PAH experts and representatives from regulatory agencies and pharmaceutical companies was convened in 2012 to discuss the validity of current endpoints, such as the 6MWD and the TTCW, as well as emerging endpoints.^8^ As clinical endpoint development is a perpetual effort, a working group continued to evaluate on clinical improvement endpoints and risk score assessment in clinical trials in PAH. There is a need for an endpoint ‘in the middle’ between ease of single endpoint 6MWD and complex endpoint TTCW; one with intermediate complexity but with greater power to demonstrate drug efficacy by objective measures and improved patient well-being.^41^ This is especially true with the multiple therapeutic options currently available, as the frequent use of combination therapies in PAH introduces additional complexities in determining effects of individual agents. A change in risk score might fulfil this need and become a comparator between treatments in phase 3 clinical trials. Moreover, the powerful influence of a risk score in predicting short-term outcomes emphasises that cohorts of patients randomised in a clinical trial should be matched on entry.

Risk scores developed based on retrospective data from registries:

#### European Society of Cardiology/European Respiratory Society risk stratification

The European Society of Cardiology (ESC)/European Respiratory Society (ERS) guidelines from 2015 and the proceedings of the 6th World Symposium on Pulmonary Hypertension from 2018 strongly recommend a regular assessment of PAH patients in expert PH centres.^42,43^ Although reliable individual predictions are difficult, patients categorised as low risk have an estimated one-year mortality rate below 5%, those with intermediate risk between 5% and 10% and those with high risk above 10%. Achieving and maintaining a low-risk profile is, therefore, the recommended goal of PAH treatment.

Any basic diagnostic programme should include at least the assessment of FC and exercise capacity, e.g. 6MWD or cardio-pulmonary exercise testing. Some further information should be obtained on right ventricular function by measuring BNP/NT-proBNP and/or by performing echocardiography and right heart catheterisation. In addition, these comprehensive risk stratification tools should be used serially to optimise outcome estimates as patients proceed through therapies.^44^

Single variables do not provide sufficient diagnostic and prognostic information. Thus, a comprehensive assessment using a risk stratification tool was developed based on 13 invasive and non-invasive variables assessed by experts in different registries as well as most commonly used at expert centres.^3,45–47^

#### SPAHR/COMPERA methodology

The prognostic advantage of a low-risk vs intermediate-risk or high-risk profile was assessed in the Swedish registry (SPAHR) of 383 assessable patients,^36^ as well as separately in the COMPERA registry including 1588 patients^35^ using eight or six variables respectively. Thereby, the discriminatory ability of the risk score assessment instrument presented in the ESC/ERS guidelines was tested. A mean risk score was calculated for each patient in the methodology of these two studies. The summated score is then used to predict five-year survival based on categorical low-, intermediate- or high-risk rankings.

#### French low-risk approach

The aim of the French Pulmonary Hypertension Registry study was to determine the association between the number of low-risk criteria present at baseline or achieved within one year of diagnosis and long-term prognosis truncated at five years using an abbreviated risk assessment tool. Risk assessment was performed according to the 2015 ESC/ERS PH guidelines.^42^ A panel of four invasive/non-invasive criteria (FC I-II, 6MWD >440 m, right artrial pressure (RAP) <8 mmHg, CI ≥2.5 L.min^–1^ per m^2^) and of three non-invasive variables only (FC I–II, 6MWD >440 m, BNP <50 ng/L or NT-proBNP <300 ng/L) was evaluated in respectively 1017 and 602 incident patients having all variables at both baseline and first follow-up.^34^

#### REVEAL scores

A US-based registry, Registry to Evaluate Early and Long-Term PAH Disease Management (REVEAL), served to develop comprehensive risk calculators. The initial REVEAL 1.0 risk score calculator with 12 variables was updated to REVEAL 2.0 with 13 variables (addition of hospitalisation within the last six months) and reassessed cut-points as well as point values. This analysis was based on a REVEAL subpopulation of 2529 patients who survived more than one year after enrolment.^37,44,48,49^ The recently published REVEAL Lite 2 score uses an abridged version of REVEAL 2.0 of six non-invasive and modifiable variables that can be implemented routinely in daily clinical practice.^50^ All three scores are used to depict one- or five-year survival as well as CW based on the individual score, change in score or categorically based on low-, intermediate- or high-risk rankings.

## Methods to enhance novel endpoint development

A composite endpoint consists of two or more component outcomes. The main advantages are that such an endpoint increases statistical efficiency by higher event rates, which may reduce sample size requirement, cost and time. Unfortunately, composite outcomes can be misleading, as studies show that treatment effects often vary, and the effect may be insignificant for the most important component and substantial for the less important components.^53^

**Fig. 1:**
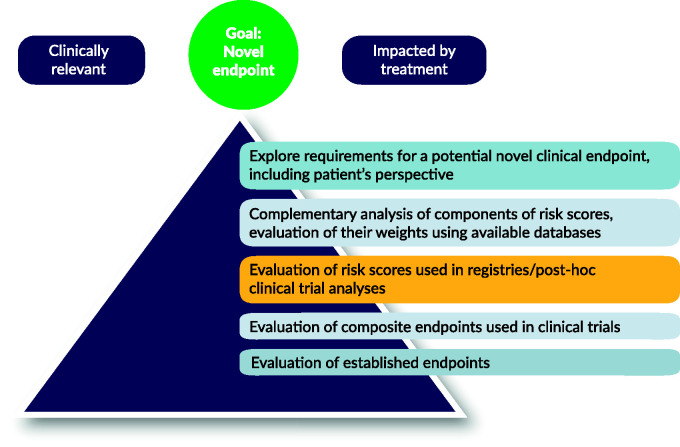
Steps to a novel composite endpoint. Source: Modified after Armstrong and Westerhout, 2017.^54^

Therefore, in order to get to novel composite endpoints, the following steps should be taken as outlined in Fig. 1:

## Potential use of composite risk score analysis as a valid clinical trial endpoint

### Retrospective post-hoc testing of risk score analysis in clinical trials

The ability of the different risk assessment tools to discriminate not only between the different risk profiles but also between treatment arms was tested post-hoc in the PATENT-1 and -2, as well as in the GRIPHON and AMBITION studies.^30,38–40^

Three abbreviated versions of this risk stratification model were previously evaluated in patients with PAH in the French, Swedish and COMPERA registries.^34–36^ These registry-derived risk scores were applied post-hoc as endpoints to randomised controlled studies such as the phase 3 riociguat PATENT database.^39^ The predictability of such scores was assessed. Six of the 13 parameters (6MWD, WHO FC, NT-proBNP, RAP, cardiac index and SvO_2_) from the ESC/ERS risk table were available and used to calculate the scores at baseline and during treatment of riociguat. Across the three abbreviated European risk scores, improvement in risk status was more pronounced under treatment, whereas more patients deteriorated in the placebo arm. The French non-invasive score, as well as the SPAHR/COMPERA score during treatment at 12 weeks of treatment, clearly discriminated long-term survival and CW-free survival.^39^

Application of the REVEAL risk score (RRS) to the PATENT study database showed similar results in terms of improvement and deterioration in favour of the verum group (riociguat); however, a considerable placebo response during treatment appeared at 12 weeks. When forming three risk strata, the improvement in risk stratum in the verum arm was statistically significant compared to placebo (*p* = 0.0181). Risk strata at baseline, under treatment at 12 weeks and change in risk stratum were significantly associated with survival and CW-free survival. The RRS predicted survival and CW-free survival over two years with hazard ratios for one-point reduction at 12 weeks of 0.705 and 0.753, respectively.^38^

These results were confirmed by a post-hoc analysis indicating the prognostic and predictive value of the French non-invasive risk assessment and the RRS 2.0 morbidity/mortality in the predominantly prevalent population of GRIPHON study, a RCT with selexipag in PAH. Patients with three low-risk criteria at baseline had a 94% reduced risk of morbidity/mortality compared to patients with no low-risk criteria and were all categorised as low-risk by REVEAL 2.0.^40^ Further post-hoc analyses available from the AMBITION study showed that improvements of 6MWD of ≥40 m increase and a drop in NT-proBNP ≥600 ng had lower risk of events (*p* < 0.01). In addition, greatest separation was seen when absolute NT-proBNP values were ≤300 ng/L. Early improvements in 6MWD and NT-proBNP predicted protection from subsequent events and may be useful to guide therapy towards long-term risk reduction.^30^

In summary, risk scores did discriminate successfully between the treatment arms and were aligned with the results from the original trials.

## Utilisation of risk stratification tools in the clinical trial arena

All the present risk assessments for PAH patients have been derived from traditional statistical methods or expert opinion. Both these and efforts to oversimplify risk-prediction have resulted in lack of robustness with respect to predicting outcomes in this complex disease. Also, clinically relevant variables, such as the rate of disease progression, currently remain unaccounted for. Not surprisingly, only a minority of patients in analysis cohorts during application of risk scores achieved the intended goal of low risk status at follow-up. Hence, the risk scores themselves need periodic refinements to incorporate new data on predictors of disease progression and mortality and, as such, to maintain their clinical utility.

There is a desire in the PAH community to incorporate risk scores into the clinical trials on PAH with the goal of using the risk score as a primary efficacy endpoint to show improvement in achieving low-risk level.^41^ TTCW should not need to be abandoned completely as an additional endpoint if intermediate and high-risk patients are enrolled into the clinical trial. Continued use of composite risk strategies will be important for future trials. In order to do this, the scores with the highest discrimination must be utilised so that investigators are confident in the simulated or predicted clinical outcome.

While risk scores have been validated in registry data or in retrospective analyses of selected clinical trials and are associated with long-term outcomes, such as mortality, the use of risk scores as a study endpoint for prospective clinical trials remains controversial.^41^

Any risk score proposed as a primary efficacy endpoint in registration trials must be sensitive to differentiate the treatment effects. The best risk score as a prognostic tool may not be the best study endpoint for prospective clinical trials. A risk score, such as REVEAL 2.0, containing both the modifiable and non-modifiable variables may be a good option as a prognostic tool, but a simplified version, such as REVEAL Lite 2,^50^ containing only the modifiable parameters may be more sensitive in differentiating the treatment effect and, therefore, be a better study endpoint. In addition, in terms of applying either of the ESC/ERS risk scores, using the total number of low-risk variables as opposed to calculating a mean score seems preferential.^33^

The available risk stratification tools vary in important ways. These include their precision, the nature of their derivation and applicability to varied subsets of PAH. The extent of validation, utility for serial use and modifiability of data elements are also variable. It has been said that experts in PAH management do not need a risk stratification tool, since their clinical expertise allows them to consider patient-specific factors in the frame of evidence-based prognostic markers. Simple use of gestalt as a holistic integrated risk system can be integrated with patient preferences and goals of therapy and lead to appropriate therapeutic decisions. Such a statement is in a sense circular, since knowledge regarding the predictive value of the tools regarding patient outcome feeds back to the astute clinician to improve their clinical acumen. Furthermore, objective results of a tool can provide a specific trigger for decision-making, such as addition of PAH therapy or referral for lung transplant and can be considered as an endpoint in clinical trials since the results may serve as a surrogate for survival and risk of hospitalisation. In addition, achieving a particular risk category based upon a tool can provide the basis for evidence-based algorithms in guidelines and, in clinical practice, be considered as a quality of care metric.

It is important to follow the recommendations from the 2014 FDA report *Voice of the PAH patient*, reminding us all that patient-reported outcomes are important and valid endpoints to consider. Day-by-day exercise capacity, symptom control and health-related quality of life are all important outcome measures valued by patients FDA.^5^

## Discussion

The myriad choices of clinical therapeutics now available have shifted our drug development models from short-term randomised placebo-controlled trials on monotherapy with a 6MWD test endpoint to trials of longer duration, with patients on multiple therapies, and primary event-driven endpoints. Nearly all pivotal studies evaluated a TTCW composite endpoint as a secondary outcome to alleviate some of the misgivings of the walk test. However, as the endpoint has varied between trials and patients enrolled are not as homogenous or their PAH as advanced, novel endpoints to demonstrate clinical outcomes and improve trial efficiency are needed. Moreover, there is a growing need from viewpoints of patient, regulators and physician scientist to develop an endpoint that reflects an improvement in health and quality of life, a delay in worsening.^55^

Early in development, investigators will need to determine which patients they wish to target with their novel therapeutic approach. Whether it is by genetic, biomarker, PAH aetiology or overall risk assessment categories, trials can be enriched. For example, the use of a biomarker known to be more prevalent or more abnormal in certain PAH subtypes could help enrol potential patients. Use of risk-prediction models that determine low-, intermediate-, high-risk populations can help streamline patients with the greatest potential benefit and, on the opposite side, those who are less advanced and may need a different primary endpoint to demonstrate efficacy.^56^ Those who are intermediate risk have potential for improvement and worsening and may be the target of choice, especially as these are usually the most prevalent group. In addition, a change in risk could be an endpoint onto itself. Utilising risk assessment scores in current early drug development trials will help validating this concept for future use in phase 3 approval studies.

Composite endpoints with equally clinically relevant components may represent a more comprehensive reflection of clinically meaningful treatment effects^8^ and should be further explored.^41^ Indeed, a new composite improvement endpoint is needed for shorter and combination therapy studies.^23^

A change in a risk score that utilises variables that change with therapy (modifiable) with a high degree of discrimination could be used as a composite endpoint.

Use of composite improvement endpoints allow individual responders to be identified, lowers the placebo response and thereby also potentially lowers the number of patients needed. PAH patients already on dual or triple therapy randomised to the control group (or placebo group) may also have a good response rate and might require a sample size similar to that in a TTCW study unless there were some enrichment strategies employed.^56,57^ The detection of treatment effects in a shorter period is also desirable and these approaches may fulfil this need.

In general, it is mandatory to assess and show each component of a composite endpoint separately in addition to the overall endpoint result. This is because composite endpoints are prone to bias in the case of component imbalances between the study groups; the worst scenario would be a positive response but higher mortality for a treatment arm. This is a potential issue with every composite endpoint and thus equally applies to endpoints of TTCW, for which definition and components varied widely between different clinical studies. The question is whether such a composite improvement endpoint is also meaningful to patients.

Post-hoc analyses of composite endpoints, such as those of the REPLACE endpoint to the riociguat PATENT studies, suggest that differences between treatment groups in clinical studies can be shown, and that these differences may be related to long-term outcomes; therefore methodological limitations of post-hoc analyses need to be taken into account.^33^

Recent composite endpoints looking at changes of parameters may improve the differentiation between treatment groups as shown in clinical trials but clinical relevance for long-term outcome needs to be further proven.

Prognostic relevance for clinical outcome is important for each of the components of a composite endpoint as well as a risk score if they are applied as a study endpoint. A retrospective analysis of clinical trial data indicates that the registry-derived risk scores may be a good practical clinical endpoint in addition to a clinical trial. While risk scores reflect the course of the disease, they do not predict molecular pathways or relevant biological processes. Risk scores may allow the early detection of disease progression. When designing a clinical trial using composite endpoint of clinical improvement or risk score as primary efficacy endpoint, we envisage that a clinical trial with risk score as primary efficacy endpoint will be designed as randomised, controlled, parallel, longitudinal design with fixed treatment duration between 12 and 52 weeks. The study endpoint could include percentage of subjects demonstrating clinical improvement, percentage of subjects achieving the low-risk criteria, absolute change in risk score or percentage of subjects with shift in risk categories (low, intermediate, high risks).

At this point, the available evidence from clinical studies suggests that composite endpoints are better validated than risk scores for application to clinical trials. It is, however, clear that in any future randomised clinical trial, the two treatment arms should be matched at baseline for risk given the powerful influence this has on outcomes.

## Conclusions

Composite endpoints used in clinical trials have led to drug approval by regulatory agencies. The components of the composite endpoints have been chosen and modified based on research from registries on clinically and outcome relevant components. Appropriately designed and validated composite endpoints can show clinically relevant changes between treatment arms in clinical trials; however, differences should be associated with long-term clinical outcomes.

Implementation of novel composite endpoints in pivotal clinical trials will require consensus between PH experts, pharmaceutical companies and regulatory authorities as well as health technology assessment bodies. The endpoints chosen should be meaningful to patients. Novel composite endpoints could be further explored post-hoc with existing pivotal clinical study databases. A next step could be the application to combined clinical study databases with larger numbers of cases. This would also increase the knowledge about the relevance and impact of different components of composite endpoints.

**Fig. 2. fig2-2045894020962960:**
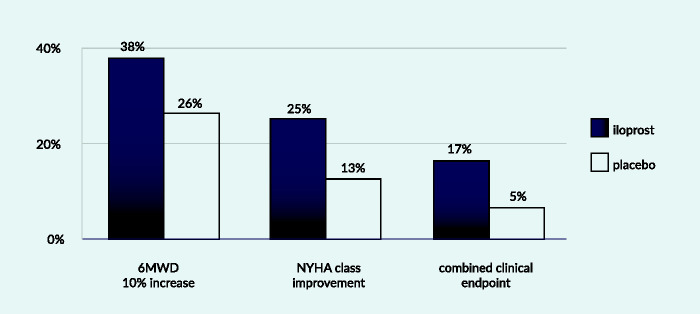
Components of composite endpoint in AIR study. 6MWD: six-minute walking distance; NYHA: York Heart Association. Source: based on Olschewski et al., 2002.^29^

**Fig. 3. fig3-2045894020962960:**
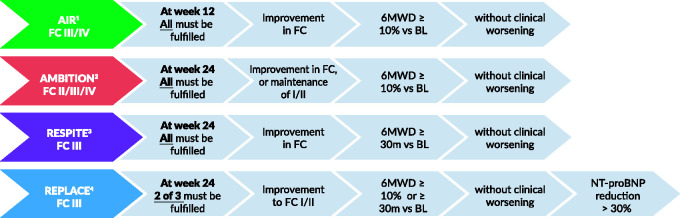
Components of combined clinical endpoints in various studies. ^1^AIR study: Olschewski et al., 2002,^29^ ^2^AMBITION study: Galie et al., 2015,^19^ ^3^RESPITE study: Hoeper et al., 2017,^31^ ^4^REPLACE study: Hoeper et al., 2017,32 and Simonneau et al., 2019,^33^ FC: functional class; 6MWD: six-minute walking distance.

**Fig. 4. fig4-2045894020962960:**
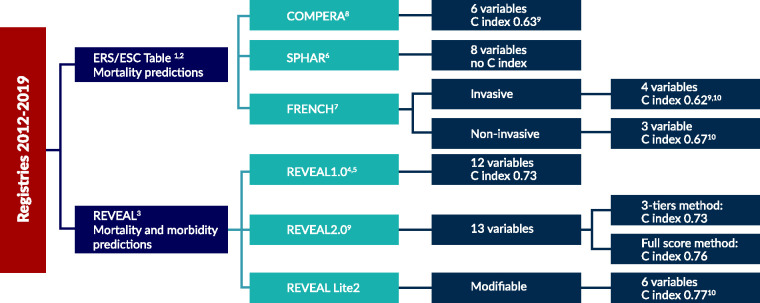
Current risk scores based on different registries and numbers of variables and discrimination indices. ^1^2015 ESC/ERS guidelines for the diagnosis and treatment of pulmonary hypertension – *Eur Heart J* 2016.^42^ ^2^2015 ESC/ERS guidelines for the diagnosis and treatment of pulmonary hypertension. *Eur Respir J* 2015.^51^ ^3^Pulmonary arterial hypertension: baseline characteristics from the REVEAL registry.^52^ ^4^The REVEAL Registry risk score calculator in patients newly diagnosed with pulmonary arterial hypertension.^48^ ^5^Prognostic implications of serial risk score assessments in patients with pulmonary arterial hypertension.^44^ ^6^A comprehensive risk stratification at early follow-up determines prognosis in pulmonary arterial hypertension.^36^ ^7^Risk assessment, prognosis and guideline implementation in pulmonary arterial hypertension.^34^ ^8^Mortality in pulmonary arterial hypertension: prediction by the 2015 European pulmonary hypertension guidelines risk stratification model.^35^ ^9^The REVEAL Risk Score Calculator 2.0 and Comparison With ESC/ERS-Based Risk Assessment Strategies.^37^ ^10^Comparison of Three Risk Assessment Strategies as Predictors of One Year Survival in US Pulmonary Arterial Hypertension (PAH) Patient.^49^ ERS: European Respiratory Society; ESC: European Society of Cardiology; REVEAL: Registry to Evaluate Early and Long-Term PAH Disease Management.

A variety of risk scores have been developed based on retrospective data from academic registries as clinically meaningful tools for the prediction of outcome in PAH patients. Risk scores are applied in clinical practice to document if the clinical status of the patient improves with therapy. There has been no clear general preference for clinical application for one of the existing risk scores at this time. Whether, and how, risk scores can also be applied in clinical trials may warrant further investigation.[Fig fig2-2045894020962960][Fig fig3-2045894020962960][Fig fig4-2045894020962960]
